# Phenotyping heart failure by nuclear imaging of myocardial perfusion, metabolism, and molecular targets

**DOI:** 10.1093/ehjci/jead128

**Published:** 2023-06-09

**Authors:** Antti Saraste, Juhani Knuuti, Frank Bengel

**Affiliations:** Turku PET Centre, Turku University Hospital and University of Turku, Kiinamyllynkatu 4–8, 20520 Turku, Finland; Heart Center, Turku University Hospital and University of Turku, Hämeentie 11, 20520 Turku, Finland; Turku PET Centre, Turku University Hospital and University of Turku, Kiinamyllynkatu 4–8, 20520 Turku, Finland; Department of Nuclear Medicine, Hannover Medical School, Hannover, Germany

**Keywords:** fibrosis, viability, PET, SPECT, cardiac sarcoidosis, cardiac amyloidosis

## Abstract

Nuclear imaging techniques can detect and quantify pathophysiological processes underlying heart failure, complementing evaluation of cardiac structure and function with other imaging modalities. Combined imaging of myocardial perfusion and metabolism can identify left ventricle dysfunction caused by myocardial ischaemia that may be reversible after revascularization in the presence of viable myocardium. High sensitivity of nuclear imaging to detect targeted tracers has enabled assessment of various cellular and subcellular mechanisms of heart failure. Nuclear imaging of active inflammation and amyloid deposition is incorporated into clinical management algorithms of cardiac sarcoidosis and amyloidosis. Innervation imaging has well-documented prognostic value with respect to heart failure progression and arrhythmias. Emerging tracers specific for inflammation and myocardial fibrotic activity are in earlier stages of development but have demonstrated potential value in early characterization of the response to myocardial injury and prediction of adverse left ventricular remodelling. Early detection of disease activity is a key for transition from broad medical treatment of clinically overt heart failure towards a personalized approach aimed at supporting repair and preventing progressive failure. This review outlines the current status of nuclear imaging in phenotyping heart failure and combines it with discussion on novel developments.

## Graphical Abstract

**Graphical Abstract:**
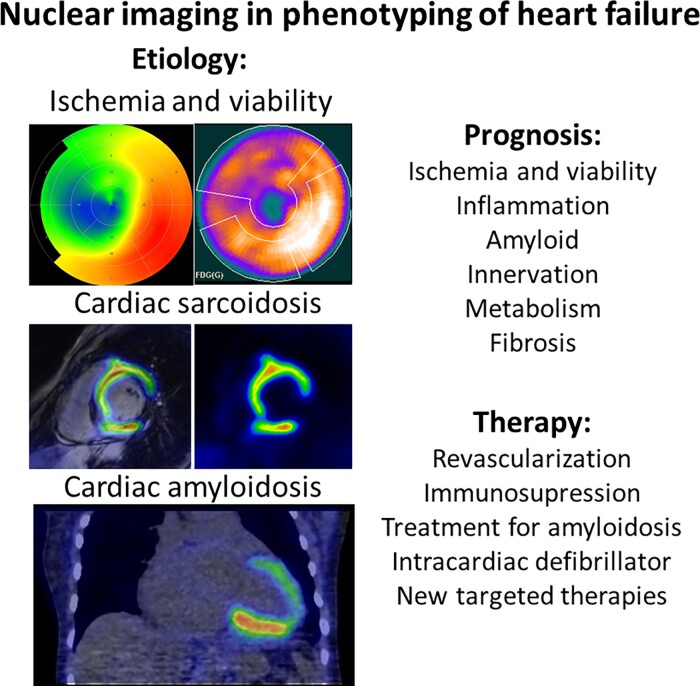

## Introduction

Cardiac imaging is essential in establishing the diagnosis of heart failure, determining its mechanisms and prognosis, identifying the specific causes of cardiac dysfunction as well as planning and monitoring treatment.^1^ Nuclear imaging techniques, single photon emission computed tomography (SPECT) and positron emission tomography (PET), allow for the detection of intravenously injected radiopharmaceuticals in tissues with very high sensitivity. Thus, nuclear imaging can provide functional and molecular information about pathophysiological processes underlying heart failure that complements assessment of cardiac structure and function with the use of other imaging modalities.^2–4^

The most common application of nuclear imaging in heart failure is evaluation of inducible myocardial ischaemia and viability to assess ischaemic aetiology of cardiac dysfunction and to predict the potential for recovery after revascularization.^1^ Yet, molecule-targeted radiopharmaceuticals enable studying several biological processes involved in heart failure beyond perfusion, including myocardial metabolism, innervation, inflammation, amyloidosis, and fibrosis.^3,4^ Currently, molecular imaging is routinely performed for the clinical diagnosis of cardiac sarcoidosis, cardiac amyloidosis as well as device or prosthetic valve infections.^5–7^ However, several emerging radiotracers and applications are in earlier stages of preclinical and clinical development for phenotyping heart failure with potential for early detection, improved risk stratification and guiding therapy in individual patients.^2–4^ This review outlines main applications of nuclear imaging in phenotyping heart failure together with discussion about novel developments.

### Technical aspects of nuclear imaging

Introduction of new radiotracers for SPECT and PET is central for advancing the role of molecular imaging in a personalized approach towards cardiovascular diseases and heart failure.^3,4^ Hybrid devices and image processing techniques facilitate fusion of PET images with high resolution morphologic images of cardiac structure and function obtained with other imaging modalities.^8^ This is particularly important for localization and quantification of tracers used for molecular imaging of small targets. Both SPECT and PET scanners are increasingly integrated with either computed tomography (CT) or cardiac magnetic resonance (CMR) systems. The use of state-of-the-art methodology enables integration of CT and nuclear imaging with an acceptable radiation exposure to the patient.^8^

Myocardial perfusion SPECT has been the mainstay of cardiac nuclear imaging as the most common non-invasive functional imaging method to evaluate suspected coronary artery disease (CAD).^9^ Recent advances in cardiac SPECT technology include developments of detector technology (solid-state detectors and cardio centric collimators), image reconstruction algorithms and dedicated cardiac scanner systems.^10,11^ As a result, image quality and resolution have improved, acquisition times have shortened and radiation exposure to the patient has reduced.^10,11^ Studies have shown the feasibility of quantification of myocardial blood flow (MBF) with the use of dynamic SPECT imaging with dedicated cardiac systems.^12,13^ Furthermore, protocols using simultaneous acquisition of multiple tracers are feasible, potentially improving accuracy of cardiac molecular imaging.^14^

Cardiac PET is an advanced nuclear imaging technique with high resolution, sensitivity, and accurate quantification of radioactivity.^15^ With the use of dynamic PET, it is possible to map myocardial radiotracer concentration and kinetics in the myocardium over time, enabling absolute quantification of many parameters, such as MBF and myocardial oxygen consumption.^16,17^ Advances in PET technology include introduction of PET scanners with long axial field of view that enable large anatomical coverage (whole-body PET) and increase in system sensitivity.^18,19^ Simultaneous assessment of multiple organs provides new possibilities for studying interactions between the heart and other organs, such as the immune system, haematopoietic system, brain, liver, and kidneys. High sensitivity combined with improved resolution provided by motion correction techniques to reduce artefacts in cardiac PET data is needed in the analysis of signals coming from small targets, such as the coronary arteries.^20,21^ The availability of PET has improved due to increase in the number of PET scanners and cyclotrons needed to produce tracers, as well as new radiotracers labelled with isotopes with half-life long enough to allow for distribution to sites without a cyclotron.^3,22,23^

### Myocardial ischaemia and viability

Ischaemic heart disease is the most common cause of heart failure.^1^ In ischaemic heart failure, left ventricular dysfunction may be reversible and improve after myocardial revascularization.^24^ Myocardial perfusion imaging with either SPECT or PET is an established modality for the evaluation of the presence and severity of CAD, contributing to the detection of ischaemic aetiology of heart failure.^1,25^ Furthermore, perfusion imaging and combined metabolic imaging can detect ischaemic myocardium that is dysfunctional, but viable and has potential for recovery of contractile function upon revascularization.^24^

Myocardial perfusion SPECT is performed with technetium-99 m (^99m^Tc) labelled radiopharmaceuticals (^99m^Tc-sestamibi and ^99m^Tc-tetrofosmin), whereas myocardial perfusion PET is mainly performed with three flow tracers: rubidium-82 (^82^Rb), 13N-labeled ammonia (^13^N-ammonia), and ^15^O-labeled water (^15^O-water).^3,16^ Short radioactive half-life of perfusion tracers (76 s to 10 min) limits the availability PET perfusion imaging, because an on-site cyclotron or a generator is needed. Perfusion tracers labelled with ^18^F, which has longer half-life (110 min), have been designed in order to enable delivery to sites without a cyclotron.^13^ Of the ^18^F-based PET perfusion radiotracers, ^18^F-Flurpiridaz showed improved sensitivity and accuracy for the detection of obstructive CAD (>50% stenosis) in comparison to SPECT in a recent Phase III study.^26,27^

Myocardial perfusion imaging detects flow-limiting coronary stenosis defined as abnormal fractional flow reserve (FFR) with high accuracy.^28^ In a recent meta-analysis comparing diagnostic methods for CAD, PET showed sensitivity and specificity of 90% and 85%, respectively, and SPECT showed sensitivity and specificity of 73% and 83%, respectively.^28^ The presence of known CAD and previous myocardial infarction may be associated with lower accuracy.^29^ Higher diagnostic accuracy of PET has been attributed to higher resolution of images, superior tracer kinetic properties and possibility to quantify absolute MBF and myocardial flow reserve (MFR, the ratio of MBF during vasodilator stress and at rest).^16,30^ Quantification of MBF provides improved characterization of the extent and severity of ischaemia in multi-vessel disease, such as balanced decreases of MBF in all major coronary artery vascular territories.^16^ Furthermore, reduced MFR detects coronary microvascular dysfunction in the absence of epicardial CAD.^16^ Several studies have demonstrated that regional perfusion abnormalities^31^ as well as impaired MFR^32^ are strong independent predictors of increased risk of future cardiac events in patients with CAD.

Coronary microvascular dysfunction often co-exists with epicardial CAD but is also common in the absence of CAD in patients with various forms of cardiomyopathy and heart failure with either reduced or preserved ejection fraction.^33–40^ In these conditions, reduced MFR was associated with the severity of systolic and diastolic dysfunction, markers of heart failure severity and poor clinical outcomes.^33–40^ In a recent study including 1255 patients with heart failure with reduced ejection fraction who underwent ^82^Rb PET, reduced MFR and stress MBF were associated with a greater risk of all-cause mortality in both ischaemic and non-ischaemic cardiomyopathy that was incremental over clinical risk factors, left ventricular ejection fraction, and the presence of scar or ischaemia.^41^ Whether coronary microvascular dysfunction in non-ischaemic cardiomyopathy is a cause or an effect of the underlying disease process and whether it can be modified by therapies remains incompletely understood.

Detection of myocardial viability is based on uptake of myocardial perfusion tracers at rest being dependent on cell viability.^24^ Furthermore, the use of specific imaging protocols, such as administration of nitrate before tracer injection or assessment of residual myocardial glucose uptake with ^18^F-fluorodeoxyglucose (^18^F-FDG) PET, can reveal viable myocardium in areas of reduced perfusion.^24,42^ A preserved or increased uptake of ^18^F-FDG in the presence of reduced resting myocardial perfusion, known as flow-metabolism mismatch, showed sensitivity of 92% and specificity of 63% for regional functional recovery after revascularization in a pooled analysis, including 24 studies with a total of 756 patients, who underwent ^18^F-FDG PET.^42^

The value of viability imaging in guiding revascularization was evaluated in a meta-analysis, including 24 single-centre observational studies with a total of 573 patients with CAD and left ventricular systolic dysfunction (mean ejection fraction 33%), showing that the presence of myocardial viability predicted a large and significant benefit from myocardial revascularization (annual mortality 16% with conservative therapy and 3.2% with myocardial revascularization).^43^ Another observational study found significant interaction with the presence of hibernating myocardium and survival benefit from revascularization, particularly when the extent of hibernating myocardium was extensive (>10% of the left ventricular myocardium).^44^ However, the randomized Positron Emission Tomography and Recovery Following Revascularization (PARR-2) trial that assigned 430 heart failure patients with an ejection fraction below 35% to either management assisted by ^18^F-FDG PET imaging or standard care, showed only a non-significant trend toward reduction in cardiac events for ^18^F-FDG PET assisted management vs. standard care.^45^ Survival benefit for ^18^F-FDG PET assisted management was found only in the subgroup of patients whose treatment adhered to the recommendations by imaging.^46^ The overall results of PARR-2 are in line with analyses in other randomized controlled trials, including Surgical Treatment for Ischemic Heart Failure (STICH), Revascularization for Ischemic Ventricular Dysfunction (REVIVED-BCIS2R), and Heart Failure Revascularization Trial (HEART), in that neither the extent of myocardial viability nor the extent of left ventricular inducible ischaemia evaluated with various imaging modalities appeared to interact with treatment strategies in favourably affecting outcomes in ischaemic cardiomyopathy.^47–50^

**Figure 1:**
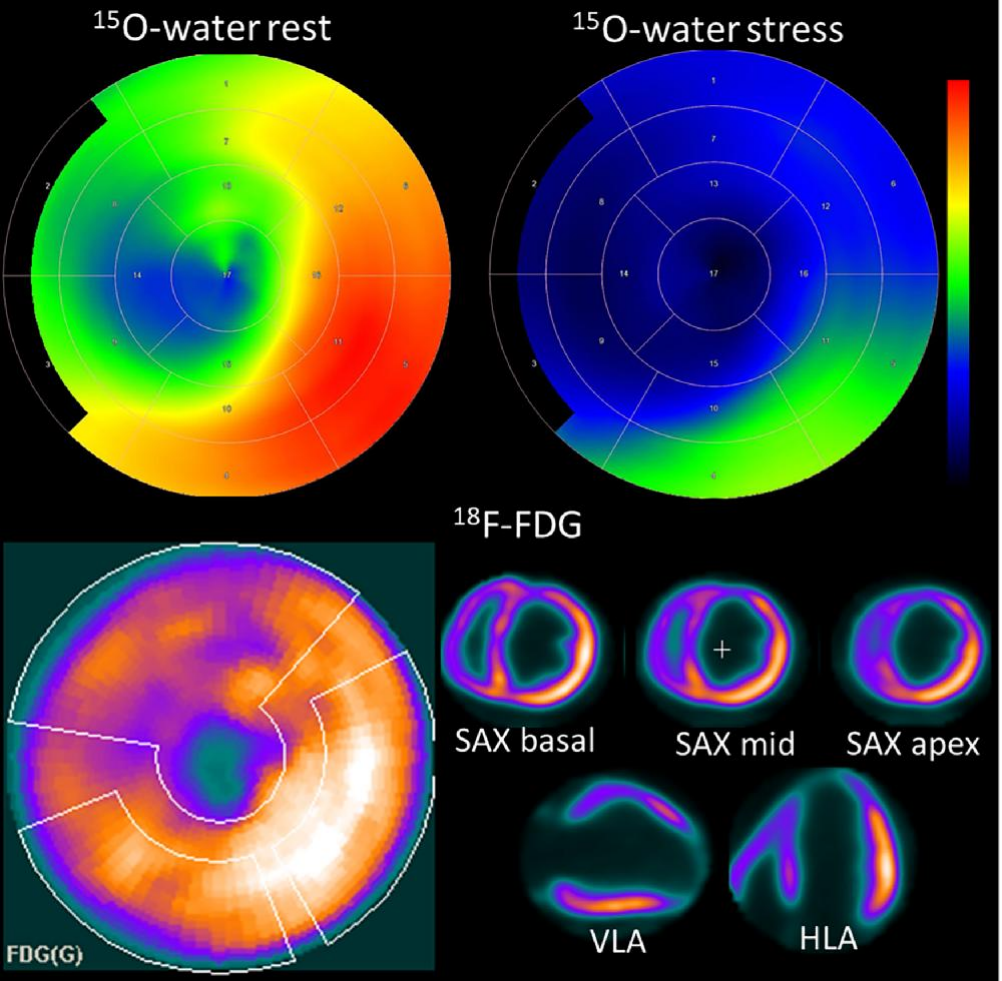
Evaluation of myocardial ischaemia and viability in ischaemic heart failure. The patient had 3-vessel obstructive coronary artery disease, severe wall motion abnormality in the territory of the left anterior descending (LAD) coronary artery, reduced left ventricular ejection fraction (35%), and high surgical risk due to co-morbidities. ^15^O-water PET showed globally reduced stress myocardial blood flow (scale is 0–3.5 mL/g/min). Resting myocardial blood flow (scale 0–1.0 mL/g/min) was also reduced in the LAD territory. ^18^F-FDG PET showed the absence of glucose metabolism in the apex indicating scar but partially preserved glucose metabolism elsewhere in the LAD territory indicating the presence of viable myocardium. The patient underwent successful coronary artery bypass surgery and ejection fraction was improved (45%) at follow-up. HLA, horizontal long axis; SA, short axis; VLA, vertical long axis.

Current guidelines recommend that myocardial revascularization may be considered in patients with chronic ischaemic heart failure with reduced ejection fraction after careful evaluation of the individual risk to benefit ratio.^1^ Although the role of routine testing of myocardial ischaemia and viability in ischaemic cardiomyopathy is controversial, it remains an option among comprehensive evaluation of heart failure patients in order to predict the response to revascularization in selected patients with CAD, in whom increased perioperative risk and the absence of typical angina makes decisions on revascularization difficult (*Figure 1*).^1,50,51^

### Inflammatory cardiomyopathy and cardiac sarcoidosis

Cardiac sarcoidosis is a granulomatous inflammatory disease with unknown aetiology, which can occur in the presence or absence of systemic sarcoidosis.^52^ In a majority of patients, cardiac sarcoidosis appears to be a slowly progressive cardiomyopathy,^53^ but ventricular arrhythmias and sudden cardiac death are common.^54^

**Figure 2:**
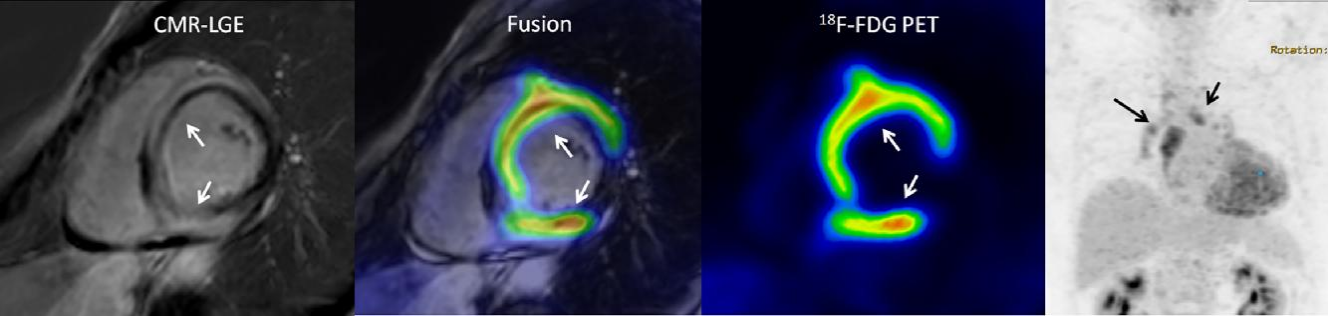
Evaluation of cardiac sarcoidosis. The patient presented with syncope, ventricular arrhythmias and reduced left ventricular ejection fraction (35%). CMR showed late gadolinium enhancement (LGE) in the interventricular septum and inferior wall. After overnight fast and low-carbohydrate diet for 24 h to suppress myocardial glucose metabolism, ^18^F-FDG PET showed marked, focally increased myocardial glucose metabolism co-localizing with LGE consistent with myocardial inflammation (arrows). In addition, ^18^F-FDG uptake was present in mediastinal lymph nodes (arrows). Granulomatous inflammation consistent with cardiac sarcoidosis was confirmed in histological analysis of an endomyocardial biopsy.

Diagnosis of cardiac sarcoidosis and stratifying the risk of ventricular arrhythmias is challenging.^52,54^ Endomyocardial biopsy is the gold standard for diagnosis, but it has low sensitivity because of the focal nature of the disease.^52^ Advanced non-invasive cardiac imaging, including CMR and ^18^F-FDG PET, play an important role in defining myocardial abnormalities in suspected cardiac sarcoidosis.^5^ While CMR provides high resolution for identifying the presence and extent of myocardial damage, ^18^F-FDG PET is a marker of active myocardial inflammation and can reveal extra-cardiac sarcoid lesions that may be targeted for histological diagnosis (*Figure 2*).^5,55–57^

Active inflammation in cardiac sarcoidosis shows as focal myocardial uptake of ^18^F-FDG, attributed to the high metabolic activity of immune cells, after suppression of physiological myocardial glucose metabolism (*Figure 2*). In a recent meta-analysis of 26 diagnostic studies (1363 patients, 528 with cardiac sarcoidosis), ^18^F-FDG PET had overall sensitivity and specificity of 82% and 82%, respectively, for cardiac sarcoidosis.^57^ However, sensitivity was 94% in studies that excluded patients who were already treated with anti-inflammatory therapy before imaging.^57^ Myocardial perfusion imaging is often combined with ^18^FDG-PET imaging to identify areas of scar, which commonly coexist with inflammation in cardiac sarcoidosis. The findings of PET have prognostic implications in patients with suspected cardiac sarcoidosis. In a series of 118 patients with known or suspected cardiac sarcoidosis who underwent ^82^Rb perfusion and ^18^F-FDG PET imaging, abnormal perfusion and ^18^F-FDG uptake on PET imaging predicted 2.9-times increased risk of ventricular tachycardia and death compared with normal PET results during follow-up.^58^

Patient preparation to suppress physiological glucose uptake is essential for the detection of cardiac sarcoidosis by ^18^F-FDG PET, since both normal myocytes and inflammatory cells take up glucose. Protocols to suppress physiological myocyte ^18^F-FDG uptake vary among studies, including prolonged fasting, low-carbohydrate meals, and/or intravenous heparin.^59^ The success of these strategies is variable, and incomplete suppression of physiological myocardial ^18^F-FDG uptake may significantly impair diagnostic accuracy of PET and in particular, monitoring disease activity in repeated studies.^5,59^ Therefore, new tracers have been sought to improve the accuracy of PET imaging in cardiac sarcoidosis. Small clinical studies and experimental studies have demonstrated promising results with ^68^Ga-DOTANOC or ^68^Ga-DOTATOC targeting somatostatin receptors on immune cells,^60,61^ radiolabelled amino acid ^11^C-Methionine PET,^62^ proliferation marker ^18^F-fluorothymidine (FLT),^63,64^ and a tracer targeting Folate receptor β expressed on activated macrophages.^65^

### Cardiac amyloidosis

Cardiac amyloidosis results from the myocardial extracellular deposition of misfolded proteins, known as amyloid fibrils, associated with left ventricular hypertrophy.^6^ Although a rare disease, recent data suggest that cardiac amyloidosis is underappreciated as an underlying mechanism of heart failure with preserved ejection fraction and hypertrophic cardiomyopathy.^66^ The introduction of targeted therapies for cardiac amyloidosis has emphasized the need for early diagnosis as discussed elsewhere in this issue.^6,66^

Bone avid SPECT radiotracers, ^99m^Tc-pyrophosphate (PYP), ^99m^Tc-3,3-diphosphono-1,2-propanodiacarboxylic acid (DPD), and ^99m^Tc-hydroxymethylene diphosphonate (HMDP) bind to microcalcification associated with transthyretin fibrils and are used to diagnose cardiac transthyretin amyloidosis (ATTR). Scans are analysed semi-quantitatively or by visual grading, myocardial uptake of the tracer equal or higher than in the ribs (Grades 2 and 3) indicating the presence of ATTR.^67,68^ In a multi-centre study of patients with suspected amyloid cardiomyopathy, significant cardiac uptake was shown to have a sensitivity close to 100% and provided that light-chain amyloidosis (AL) was excluded by plasma or urine testing, a specificity and positive predictive value close to 100% for the diagnosis of ATTR.^69^ In some hereditary forms of the disease, sensitivity of SPECT scintigraphy may, however, be lower.^70,71^ Semi-quantitative markers of cardiac tracer uptake correlate with markers of disease severity and mortality.^68^ However, quantitative measures of amyloid burden are sought to better stratify prognosis, assess subtle cardiac uptake possibly representing early stage of disease, and detect changes in amyloid burden in response to disease-modifying therapy.^68^ Small studies have found correlation between quantitative SPECT measures of PYP and DPD accumulation and left ventricular mass and myocardial extracellular volume measured by CMR indicating amyloid burden.^72,73^

Amyloid-targeted PET tracers are emerging in evaluation of suspected cardiac amyloidosis. The original ^11^C-labeled Pittsburgh compound B (^11^C-PIB),^74–76^ as well as the ^18^F-labeled alternatives florbetaben or florbetapir,^77,78^ bind to both transthyretin and light-chain amyloid and allow the detection of cardiac AL and ATTR. Amyloid-targeted PET provides quantitative measures of about amyloid burden in the myocardium and other organs, potentially already in early stages of disease.^76,79^ The cardiac uptake of ^11^C-PIB has been found to correlate with the degree of histological myocardial amyloid deposition^80^ and independently predict outcome in cardiac AL amyloidosis.^80,81^ Furthermore, quantification may be relevant for determining response to disease-modifying therapies.^74,75^

When cardiac amyloidosis is suggested clinically and supported by echocardiographic or CMR findings, current guidelines recommend SPECT bone scintigraphy for the diagnosis of ATTR.^6,65^ Provided that AL is excluded by evaluation of a monoclonal immunoglobulin light-chain clone with serum protein and urine protein electrophoresis with immunofixation, and serum-free light-chain ratio, a positive scan is diagnostic for ATTR and biopsy is not needed, and the patient can be referred to disease-modifying targeted therapy.^6,68^ Novel tracer-based imaging techniques may in the future help in monitoring the success of such therapies.

### Innervation imaging

The development of radiotracers for non-invasive imaging of cardiac innervation has provided insights into pathophysiology of cardiomyopathies and a non-invasive approach to risk stratify patients with heart failure and at risk of arrhythmias.^3,82^ The most common approach to study cardiac innervation is by radiolabelled catecholamine analogues targeting presynaptic nerve terminals, including SPECT tracer ^123^I-metaiodobenzylguanidine (^123^I-MIBG) and PET tracer ^11^C-metahydroxyephedrine (^11^C-HED).^82^ In heart failure, myocardial norepinephrine circulation becomes abnormal related to activation of the sympathetic nervous system or damage to presynaptic nerves.

Many studies have demonstrated that reduced cardiac uptake of ^123^I-MIBG is a predictor of poor prognosis in heart failure with different aetiologies.^83^ Cardiac uptake of ^123^I-MIBG is typically measured as myocardial activity relative to background mediastinal activity [heart-to-mediastinum (H/M) ratio] or myocardial washout. The prospective, multi-centre trial AdreView Myocardial Imaging for Risk Evaluation in Heart Failure (ADMIRE-HF), which included 961 patients in New York Heart Association functional Class II–III and ejection fraction ≤35% for a 17-month follow-up, showed that late H/M ratio <1.6 was associated with an increased incidence of worsening NYHA class, life-threatening arrhythmias, and cardiac death.^84,85^ A H/M ratio >1.6 was associated with 1% incidence of cardiac death per year, whereas H/M ratio <1.2 was associated with almost 10% higher incidence (9.6%). More recently, regional heterogeneity of cardiac innervation has emerged as a risk factor for ventricular arrhythmias.^82^ Studies suggest that the presence of regions of abnormal innervation with relatively preserved perfusion or viability (innervation/perfusion mismatch), typically located adjacent to scarred regions, can represent a marker of arrhythmic susceptibility in patients with CAD.^86,87^ In the Prediction of ARrhythmic Events with Positron Emission Tomography (PAREPET) trial, the extent of regional denervation assessed with quantitative ^11^C-HED PET was predictor of sudden cardiac arrest (arrhythmic death or intracardiac defibrillator shock for ventricular tachycardia >240/min or ventricular fibrillation) independently of ejection fraction, infarct volume, symptoms, and natriuretic peptide level in CAD patients who were candidates for an implantable cardioverter defibrillator placement for primary prevention of sudden cardiac death.^88^

Despite prognostic evidence of innervation imaging, in the absence of prospective evaluation of the prognostic impact of innervation imaging on clinical decision-making and patient management, it has not been incorporated in clinical practice guidelines to risk stratify heart failure patients. Yet, it might be of value in specific patient groups, such as borderline risk for arrhythmias based on other investigations, as well as in guiding therapeutic interventions, such as catheter ablation of ventricular arrhythmias.^82^

### Advanced metabolic imaging

**Figure 3:**
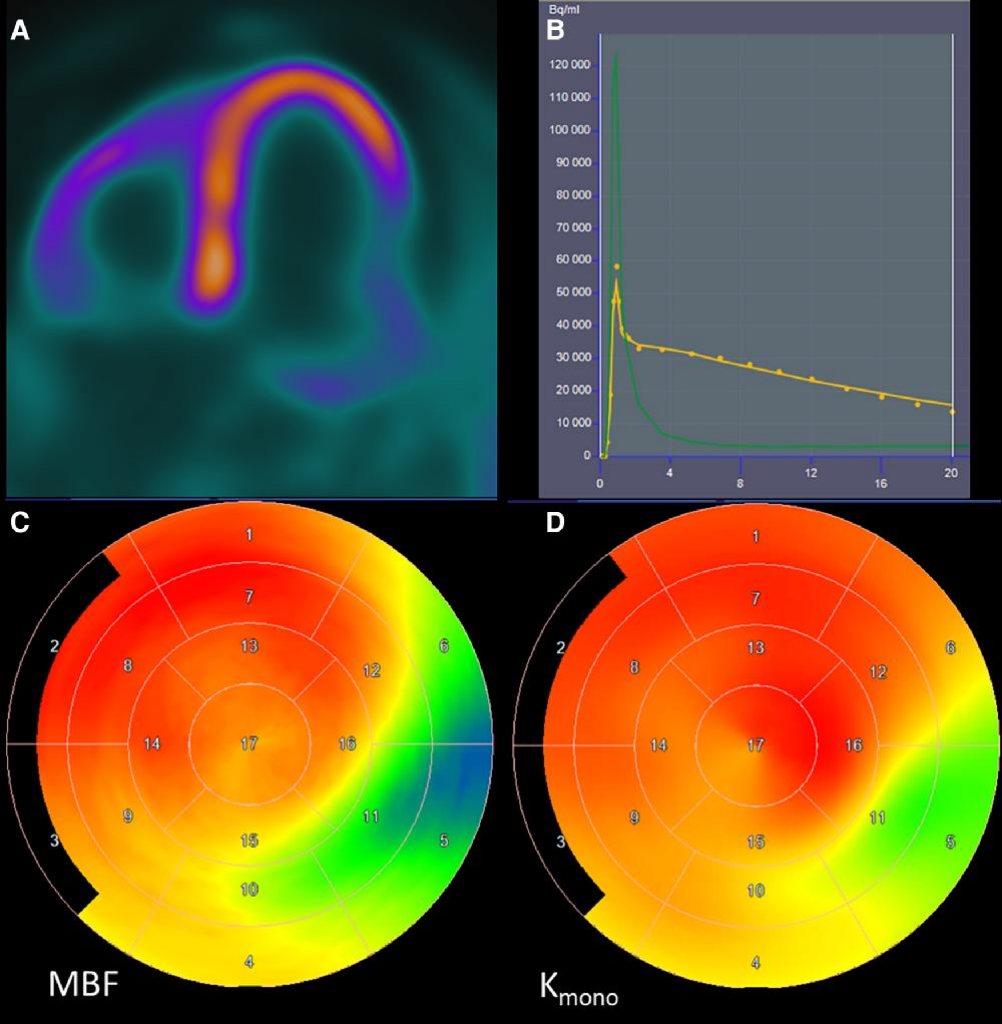
Evaluation of myocardial oxidative metabolism and efficiency of external work in a patient with ischaemic cardiomyopathy. PET image shows ^11^C-acetate accumulated in the myocardium in proportion to blood flow during the first minutes after injection (*A*). ^11^C-acetate is trapped in the myocytes as ^11^C-acetyl coenzyme A, which is metabolized in the mitochondrial tricarboxylic acid cycle, and radioactivity is cleared from the myocardium as ^11^C-carbon dioxide. The rate of ^11^C-clearance during 20 min is monoexponential (K_mono_) that is linearly related to myocardial oxygen uptake (yellow line in *B*). (*C*) Map of resting myocardial blood flow and (*D*) Kmono in the left ventricle myocardium are shown. Note reduced blood flow and Kmono in the area of infarct scar in the lateral wall. Myocardial external efficiency can be calculated as the ratio of cardiac work (cardiac output per myocardial mass times mean arterial pressure) and myocardial oxygen uptake. Cardiac output can be measured by ultrasound, cardiac magnetic resonance, or a recently developed method based on radioactivity concentration in the left ventricle cavity.^91^

Metabolic PET tracers enable studying myocardial oxygen consumption and substrate metabolism beyond glucose utilization.^17,89,90^ Myocardial external efficiency describes the ability of myocardium to convert energy into mechanical work.^17^ This can be evaluated non-invasively as the relation of left ventricular external work (product of mean arterial pressure and stroke volume) and myocardial oxygen uptake measured by carbon-11 acetate (^11^C-acetate) PET (*Figure 3*).^17,92^ In order to analyse myocardial substrate metabolism, glucose uptake assessed with ^18^F-FDG can be measured quantitatively and combined with tracers of fatty acid metabolism, such as fatty acid analogue 18F-fluoro-6-thia-heptadecanoicacid (^18^F-FTHA) or carbon-11 labelled palmitate (^11^C-palmitate).^89,90^ Studies using metabolic PET tracers have shown distinct alterations in myocardial substrate metabolism and myocardial external efficiency in heart failure that are related to alterations in systemic metabolism, such as those that occur in obesity and diabetes.^89,90^

Reduced myocardial external efficiency is a typical feature of heart failure with reduced ejection fraction.^93–95^ Furthermore, it may be reduced in the presence of relatively preserved ejection fraction as shown in patients with hypertrophic cardiomyopathy,^96^ cardiac amyloidosis,^97^ and severe valvular heart disease.^91,95^ Myocardial efficiency is directly associated with systolic dysfunction and inversely related to afterload, activation of the sympathetic nervous system, increased wall stress and left ventricular hypertrophy,^93,94,98^ the contributions of each varying depending on aetiology, and stage of the underlying cardiac disease. Although myocardial oxygen consumption may be similar or even reduced in heart failure when compared with healthy controls, it is increased in relation to mechanical work.^93,94^

There is currently relatively limited information on prognostic and therapeutic implications of imaging myocardial metabolism and efficiency in heart failure. In a small study, impaired myocardial efficiency was a stronger predictor of survival than ejection fraction in patients with dilated cardiomyopathy.^99^ Furthermore, impaired myocardial efficiency has been associated with reduced exercise capacity and severity of heart failure symptoms.^87,88,90^ However, it remains unknown to what extent reduced myocardial external efficiency represents a cause or sequence of cardiac dysfunction. Therapies that have been proven beneficial in heart failure, such as beta-blockers, mineralocorticoids, and cardiac resynchronization, have been shown to improve myocardial efficiency.^17^ However, studies that have addressed the effect of manipulating substrate metabolism have yielded discouraging results.^90^ For example, acute fatty acid deprivation by blocking lipolysis in patients with idiopathic dilated cardiomyopathy, in contrast to healthy controls, led to reduced stroke volume, but not oxidative metabolism so that myocardial efficiency deteriorated further.^100^ It remains to be seen whether increased availability of cyclotrons to produce ^11^C-acetate together with standardized analysis tools^97^ will result in increased use myocardial external efficiency as a tool to study therapeutic interventions in heart failure.

### New markers of myocardial injury and fibrosis

Acute myocardial injury, such as myocardial infarction, initiates several maladaptive changes in cardiac myocytes and the extracellular matrix, which can contribute to progressive left ventricular dysfunction, adverse remodelling, and eventual failure.^101^ Inflammation, fibrosis, and angiogenesis play a role in myocardial repair after acute myocardial injury. However, excessive inflammation may be harmful and diffuse interstitial myocardial fibrosis is a common pathophysiological pathway in different forms of heart failure, the extent of which is associated with mortality.^102^ New targeted tracers have been evaluated in experimental and translational clinical studies to evaluate the extent, time-course, and prognostic value of these mechanisms in cardiac diseases.

Myocardial inflammation following acute myocardial infarction has been studied with ^18^F-FDG^103^ and tracers targeting chemokine receptors CXCR4^104,105^ and CCR2,^106^ somatostatin receptors,^107^ the mitochondrial 18kD translocator protein TSPO,^108^ and other pro-inflammatory targets.^2^ These studies have demonstrated increased tracer uptake in the infarcted region early after myocardial infarction, indicating the immune response to myocardial injury. Of note, an overzealous response, as indicated by these imaging markers of early post-infarction inflammation, provides information about the risk of subsequent development of cardiac dysfunction, adverse remodelling, and even patient outcome that may be incremental over standard markers of scar extent.^103–105,107,108^ Molecular imaging of the immune response to injury may even be used in a ‘theranostic’ setting, for guidance of targeted immunomodulatory drug intervention. In an experimental study, a CXCR4-targeted imaging signal was used to identify optimal timing and candidates for treatment with a CXCR4-blocking drug, which subsequently improved function only when PET indicated high expression of the CXCR4 target in myocardial tissue.^104^ Other studies have also shown the potential of nuclear imaging to characterize associations between inflammatory response in the myocardium and systemic inflammatory networks,^109^ such as in the haematopoietic organs^107^ and the central nervous system.^108^ Such comprehensive, systems-based information may help even further in personalizing targeted therapies, by accounting for the status of other organs and tissues that interact with the cardiovascular system.

**Figure 4:**
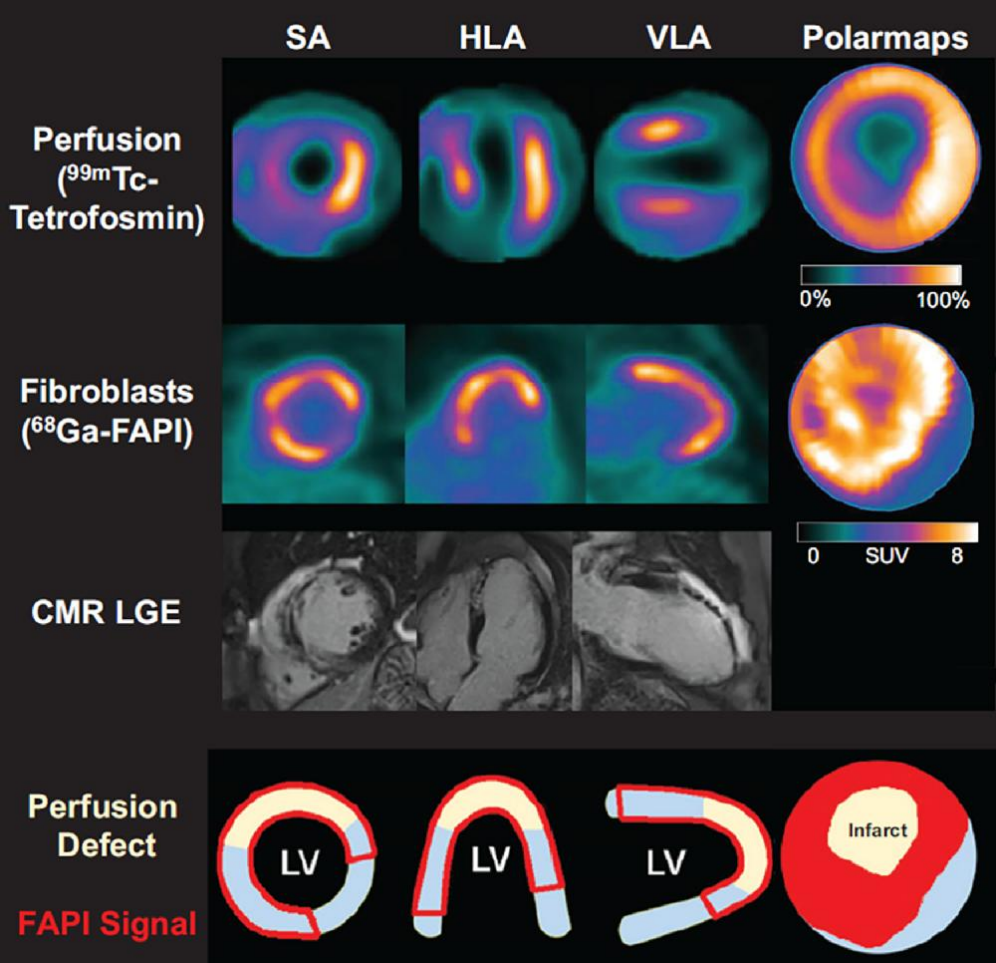
Myocardial perfusion images using 99mTc-tetrofosmin at rest, 68Ga-FAPI PET, late gadolinium enhancement (LGE) from CMR, and schematic drawings of the LV. Area of myofibroblast activation as indicated by 68Ga-FAPI-PET signal exceeds infarct area and LGE signal. HLA, horizontal long axis; SA, short axis; VLA, vertical long axis. Reproduced with permission from reference^115^.

Activation of the immune system in response to injury leads to activation of pro-fibrotic mechanisms, which initially contribute to tissue repair, but, if maladjusted, may also contribute to excessive fibrosis limiting myocardial contractile function.^101^ Hence, fibrosis and fibrotic activity have emerged as further targets for molecular therapy, and thus also for diagnostic imaging. While CMR provides quantitative measures of extracellular tissue composition, including fibrosis, via T1 and T2 mapping, nuclear imaging approaches focus on the cellular substrate for pro-fibrotic activity. Integrin receptors,^110,111^ matrix metalloproteinases,^112^ neurohormonal activation,^113^ and myofibroblast activation^114^ may be identified using targeted tracers, and thereby provide molecular signatures of the individual pro-fibrotic activity. Fibroblast activation protein (FAP), for example, is a membrane-bound serine protease highly expressed by activated myofibroblasts that can be targeted by ^68^Ga-FAPI PET. In clinical pilot studies, ^68^Ga-FAPI PET showed strong signal early after acute myocardial infarction that exceeded the infarct region indicating that FAP upregulation plays a role not only in replacement fibrosis in the primary injured region but also in reactive fibrosis that may compromise non-infarcted myocardium (*Figure 4*).^114^ Furthermore, the FAP signal was distinct from CMR tissue characterization and its extent correlated with contractile dysfunction at follow-up.^115^ Activation of fibroblasts as well as inflammation represent early steps in the initiation of myocardial fibrosis and therefore, their imaging may provide an opportunity for an early intervention at a reversible stage before irreversible fibrosis.^116^ Of note, highly specific molecular therapies are emerging that may be able to reverse interstitial fibrosis in heart failure.^117^ Such therapies may be guided by FAP-targeted PET or other imaging markers of fibrotic activity, towards the most suitable candidates and timing in the future, in a manner similar to other fields of image-guided, personalized medicine.^118,119^

## Summary and future directions

Nuclear imaging of myocardial perfusion and viability serves as an established tool for the detection of ischaemic aetiology of heart failure and therapeutic guidance before revascularization (Graphical abstract). However, the use of nuclear imaging in cardiac diseases has been changing due to advances in molecular imaging as well as advances in other imaging modalities, emerging evidence questioning established approaches to diagnose and risk stratify CAD and the emergence of specific, targeted therapies that require imaging biomarkers for guidance with respect to identifying the right patients and timing. High sensitivity of nuclear imaging together with development of new targeted tracers enables assessment of various cellular mechanisms of heart failure, such as inflammation, infiltration, innervation, metabolism, and fibrosis. Furthermore, recently introduced whole-body scanners provide improved evaluation of systemic distribution of tracers to study the interrelationships between the heart and other organs.

Nuclear imaging is now routinely used for the diagnostic evaluation of cardiac sarcoidosis and amyloidosis. Innervation imaging has well-documented potential to predict heart failure progression and risk of arrhythmias, but its specific role in patient management remains to be established and will depend on integration with guidance of therapies. New tracers, such as those specific for inflammation and detecting early activation of fibrotic processes have shown promise as biomarkers to assess functional outcomes after myocardial injury. Molecular imaging of early disease activity has potential to provide tools for transition from current practice of treating heart failure to a future of biomarker-based precision medicine aiming at prevention of heart failure and support of tissue repair. However, clinical implementation requires clear definition of the prognostic value of new imaging techniques beyond existing imaging techniques and demonstration of their ability to guide selection of therapies.

## Data Availability

No new data were generated or analysed in support of this research.
